# Internal oblique line implants in severe mandibular atrophies

**DOI:** 10.4317/jced.57675

**Published:** 2020-12-01

**Authors:** Argimiro Hernández-Suarez, Luis-Guillermo Oliveros-López, María-Ángeles Serrera-Figallo, Celia Vázquez-Pachón, Daniel Torres-Lagares, José-Luis Gutiérrez-Pérez

**Affiliations:** 1 DDS, OMS, MSc. PhD student at Dental School, University of Sevilla (Seville, Spain). Director of National Center of Oro-Maxillofacial Surgery and Implants CIBUMAXI, Caracas, Venezuela; 2 DDS, MOS. PhD student at Dental School. University of Sevilla, Seville, Spain; 3 DDS, MOM, MOS, PhD. Assistant Professor of Oral Surgery at Dental School. University of Sevilla, Seville, Spain; 4 DDS, MOS and PhD student at Dental School. University of Sevilla, Seville, Spain; 5 DDS, MOS, PhD. Full Professor of Oral Surgery at Dental School. University of Sevilla, Seville, Spain; 6 DMD, OMS, PhD. Tenure Professor of Oral Surgery at Dental School. University of Sevilla, Seville, Spain. Head of Oral and Maxillofacial Surgery Service at Virgen del Rocio University Hospital, Seville, Spain

## Abstract

**Background:**

Maxillary atrophy may be related to mechanical, inflammatory or systemic factors, being a consequence of a reduction in the amount and quality of available bone. Several surgical techniques have been developed for the restoration of bone volume needed for placing dental implants; guided bone regeneration or three-dimensional reconstructions with autologous bone, inter alia, are techniques described in the literature which demonstrate this, all of which preceded by a proper prosthetic surgical assessment. Even when the majority of authors recommend the use of these techniques prior to placing implants, it has been shown that implants with a smaller diameter and length may be placed in severely atrophied jaws without the need for performing any surgery, offering excellent results.

**Material and Methods:**

Twenty-four (24) implants were placed in six patients with severe mandibular atrophy. The implants were placed in the anterior sector and on an internal oblique line. Patients were rehabilitated with a total implant-supported prosthesis, with monitoring over a 10-year period.

**Results:**

After a 12-month monitoring period, all the patients presented successful rehabilitation. Marginal bone loss in general (n=24 implants) was +0.11 mm ± 0.53. In the implants in zones 1 and 4 (posterior) it was +0.06 mm ± 0.48 and in implants in zones 2 and 3 (anterior), +0.14 mm ± 0.57.

**Conclusions:**

Implants can be placed in the anterior zone and on an internal oblique line in patients with severe mandibular atrophy, using a diameter and length adapted to bone availability, for later prosthetic rehabilitation, offering satisfactory results since phonetic and masticatory function can be restored, as well as facial and buccal aesthetics, in a single surgical operation, with minimum morbidity.

** Key words:**Severe atrophy, implants, bone grafts, ridge atrophy, internal oblique line.

## Introduction

Currently oral surgeons must be able to solve the needs of all patients and must provide surgical management capable of solving difficult cases with optimal results. Mandibular atrophies produce an incapacitating condition in patients, due to their progression and irreversibility (1). The main cause of mandibular atrophy is tooth loss, triggered by several factors including periodontal and mechanical ones, tooth decay, etc. (1,2). It is worth stressing that age significantly affects facial disorders caused by these atrophies, since the ageing process usually exacerbates said disorders.

Atrophies alter maxillomandibular ratios and they reduce the amount of bone in the area bearing the dentition and the depth of the vestibular groove. Patients tend to experience excessive mobility of the muco-supported prosthesis, persistent ulceration and neuralgia (2).

The mandible presents a pattern of centrifugal resorption. Tooth loss gives rise to surrounding alveolar bone remodelling and resorption, eventually causing atrophic edentulous ridges (2). Bone density of maxillae also decreases after dental loss. Change in density is greater in posterior sectors and lesser in the anterior sector (2). Despite large mandibular resorption, the retromolar zone is usually maintained in optimal condition (external and internal oblique line).

Classically, (1) authors divides surgical procedures to correct alveolar atrophies into two categories. Techniques for compensating atrophies, in which procedures are included for extending the vestibule, lowering of the floor of the mouth or both, which are indicated when the ridge is affected by muscular insertions or high mucosae and techniques for correcting atrophies, in which the maxillary ridge needs to be enlarged by substituting lost bone, are the surgical procedures of choice when the bone height is not adequate.

Currently for lost bone replacement many surgical techniques and regeneration materials have been employed (3-9). One of the most common is the guided bone regeneration technique for alveolar ridge augmentation (5,6). It consists of placing membranes which act as a barrier mechanism in bone defects for promoting clot formation and preservation and preventing the migration of epithelial or connective tissue, which enables clot differentiation in bone tissue (5,6). Notwithstanding, it is difficult to provide adequate space for regeneration and obtain sufficient bone volume; this technique is more useful for limited defects of the alveolar ridge. (6) 

Although many bone augmentation techniques have been developed, autologous bone grafting continues to be the one most used in maxillofacial reconstruction (5,8-14). In order to reduce morbidity associated with autologous bone use, some authors have described how the use of frozen and desiccated allogeneic bone, freeze-drying, despite reducing the antigenicity of the allogeneic bone, alters its physical properties, which results in a reduction of osteogenic capacity, and greater resorption (7). The use of grafts in corticospongious blocks has also been described, which may be obtained from several areas, both extra-oral ones (iliac crest o cranial vault) and intra-oral ones (mandibular branch, chin, maxillary ridges) (9-12), and osteogenic distraction, a technique for gradual bone elongation which uses natural healing mechanisms for generating new bone by means of the use of a distractor (16-19).

The use of short implants is very widely used at present for solving large maxillomandibular resorption. They are a simple, quick and economic solution to bone augmentation procedures. Several studies have described that short implants with a length of 5 to 6 mm can have a similar short-term survival rate when compared to conventional implants placed in regenerated bone (20-22).

The All on four (All on 4) technique, described by Dr. Maló *et al.*, is a surgical procedure which enables immediate fixed maxillomandibular rehabilitation on 4 implants, avoiding anatomical structures and major bone regeneration surgery (23,24).

Surgical bone regeneration procedures of the maxillary bones described earlier, are not free from complications that can lead to treatment failure. Problems arising from healing, post-operative infections, neurological lesions, inter alia, can expose the graft and increase post-operative morbidity (8,10,25-27).

On reviewing the literature, countless authors referred to the advantages and disadvantages of the different techniques used to solve mandibular atrophies using bone regeneration, but all of them coincide in that when comparing them with other techniques that do not employ bone regeneration, they present a high rate of morbidity. For this reason, our aim is to present a new surgical technique for muco-supported implantological rehabilitation in completely edentulous patients, with severe mandibular atrophies, which presents a low failure rate, using the internal oblique line as an anatomical reference point.

## Material and Methods

Based on a retrospective observational study, six patients were selected who attended consultation at the bucomaxillofacial service, CIBUMAXI, in Caracas, Venezuela, over a period of 10 years, from 2006 to 2016, who presented in their clinical and imaging assessment a diagnosis of severe mandibular atrophy and having explained their treatment options, they accepted this alternative treatment (internal oblique line implants) for their oral rehabilitation.

Diagnosis of severe mandibular atrophy was obtained using an integrated assessment, in which each patient was evaluated clinically by the surgeon and prosthetist, an imaging study which included a panoramic radiograph and computerised tomography were performed, and study models were made (Pax 3D, Vatech, Korea; Software Easydent4, Vatech, Korea).

The requisite for joining the study was that patients had a diagnosis of severe mandibular atrophy, that conditions of a pre-existing systemic illness had to be controlled, that they were non-smokers and that they did not have any absolute contraindication for the placement of dental implants. The study protocol was approved by the Ethical Committee of the University of Seville. All the patients read and signed the informed consent form in order to take part in the study. The guidelines set out in the Helsinki Declaration were observed for testing on humans.

**Figure 1:**
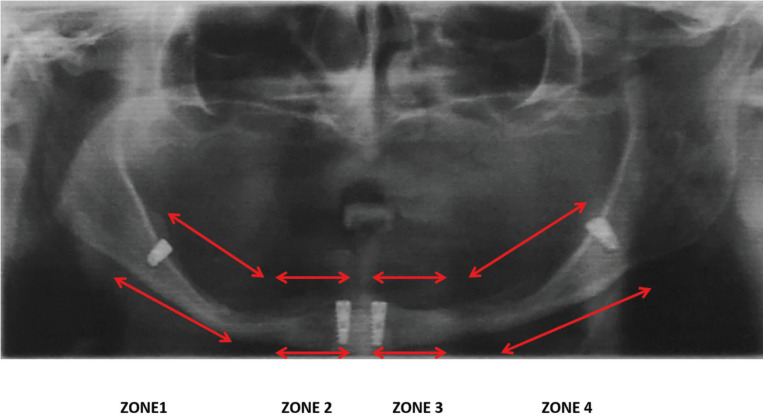
Operated zone for implant placement.

The implants were placed in four mandibular zones (zone 1, zone 2, zone 3, zone 4), zones 1 and 4 being the posterior areas, and zones 2 and 3, the anterior mandibular areas (Fig. 1). All the implants were placed using the transmucosal technique, with no bone graft or connective tissue graft, with delayed loading. The final prosthesis was placed in all cases 1.5 months after the second surgical procedure. A ball retainer was always used, of the same make as the implant that was placed as the case might be, with a metal female component and a nylon cap. All the antagonists were complete conventional prostheses and in one case an overdenture on the four dental implants placed. Patients did not use a provisional prosthesis during the osseointegration process of the implants.

-Description of the surgical technique:

After the integrated assessment of each patient, once the measures corresponding to the zones where the implants were to be placed had been carried out and the correct position, diameter and length of the implants had been determined, the surgical procedure was performed.

**Figure 2:**
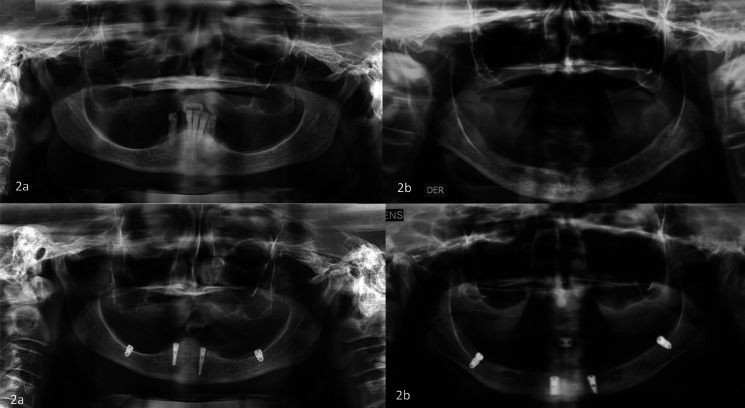
a) Radiographic image taken before and after implant placement. Clinical case. b) Radiographic image taken before and after implant placement. Clinical case.

**Figure 3:**
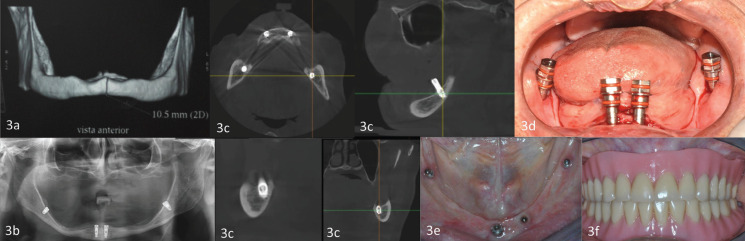
a) Tomographic image. Clinical case. b) Panoramic radiograph after implant placement. c) Tomographic image with implants in place. Clinical case. d) Clinical image of patient with implants. e) Image of implants in mouth. Clinical case. f) Image of patient with prosthesis. Clinical case.

Placement of the implants was undertaken using the transmucosal technique (without the need for making a flap), following the standard milling protocol, initially two implants in the anterior mandibular zones were placed in the lateral incisor or canine zone. After this the implants in the posterior zone (internal oblique line) were placed, taking into account the measurements and the location obtained in the prior analysis of the clinical case study, defined between the alveolar ridge and the mylohyoid line (internal oblique line) where the corresponding milling was performed penetrating the lower edge of the mandible, in search of primary bicortical stability; during the milling protocol it is advisable to maintain the index finger in the lingual zone such that correct placement of the implant can be controlled and ensured (Figs. 2,3).

It is important to highlight that there should be parallelism in the four implants to be placed and their permissible error margin is of just 200. The implants remained submerged for three and a half months, since despite having an average bone resistance of 40Nw, we must remember that they are older adult patients and they present for the most part a bone density of D3 and D4 in the anterior zone and D1, D2 in the posterior zone, on the Misch classification.(2) After the waiting period the second stage surgery was undertaken, the healing collars were placed and the prosthetic rehabilitation stage was initiated.

## Results

The number of cases selected which met the selection criteria were six patients, with a 12-month follow-up, of both sexes, with an average age of 70 years, with controlled systemic conditions, non-smokers and who accepted this treatment proposal. All the implants were internal hexagonal platform connection, SLA surface, and made of ELI grade V titanium, although of different brands (AB-Ashdod, Israel) (MIS-Or Yehuda, Israel) (AlphaBio-Tel Aviv, Israel) (Neobiotech-Seoul, Korea) (Table 1). The bone was always type 1, except in three posterior zones of three different patients in which it was type 2. Osseointegration time was 3.5 months in all cases. The prosthesis retention ball used the same brands as the implant used in each case (AB-Ashdod, Israel) (MIS-Or Yehuda, Israel) (AlphaBio-Tel Aviv, Israel) (Neobiotech-Seoul, Korea).

**Table 1:**
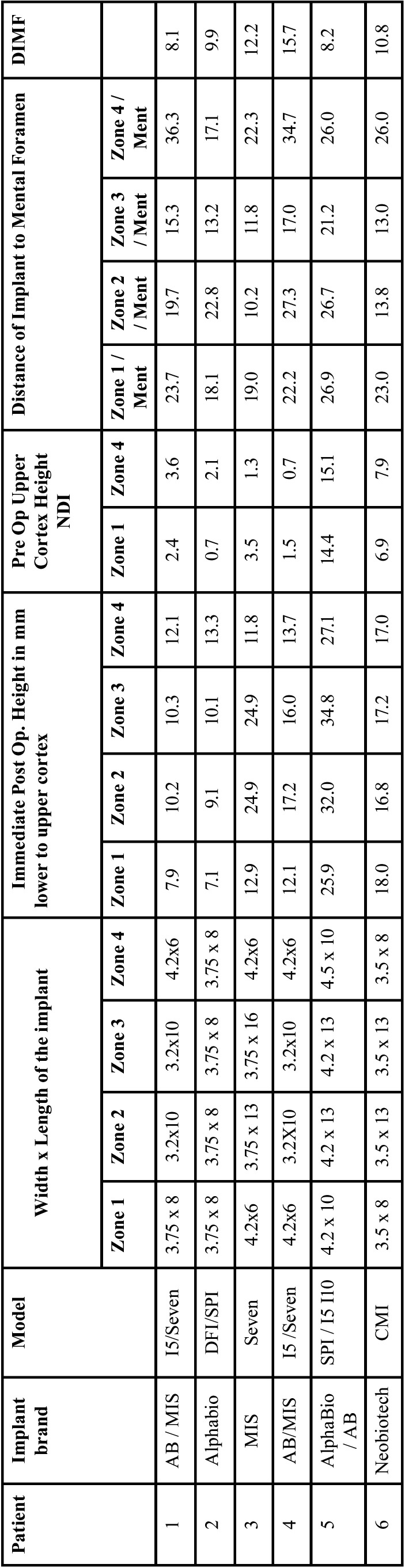
Types of implants used and immediate post-operative results in operated zones.

Regarding the implants used, in zones 1 and 4, the mean thickness was 3.995 mm ± 0.32 and the mean length was 7.5 mm ± 1.50. In zones 2 and 3, the mean thickness of the implants used was 3.6 mm ± 0.36 and the mean length was 11.41 mm ± 2.4 (Table 1).

After a 12-month follow-up, the bone change in general (n=24 implants) was +0.11 mm ± 0.53. In the implants in zones 1 and 4 (posterior) it was +0.06 mm ± 0.48 and in implants in zones 2 and 3 (anterior), +0.14 mm ± 0.57 (Table 2). After 12 months the only failure of the series occurred (survival 95.83%). It was an implant placed in zone 4, which was not replaced, and the prosthesis has continued to function correctly.

**Table 2:**
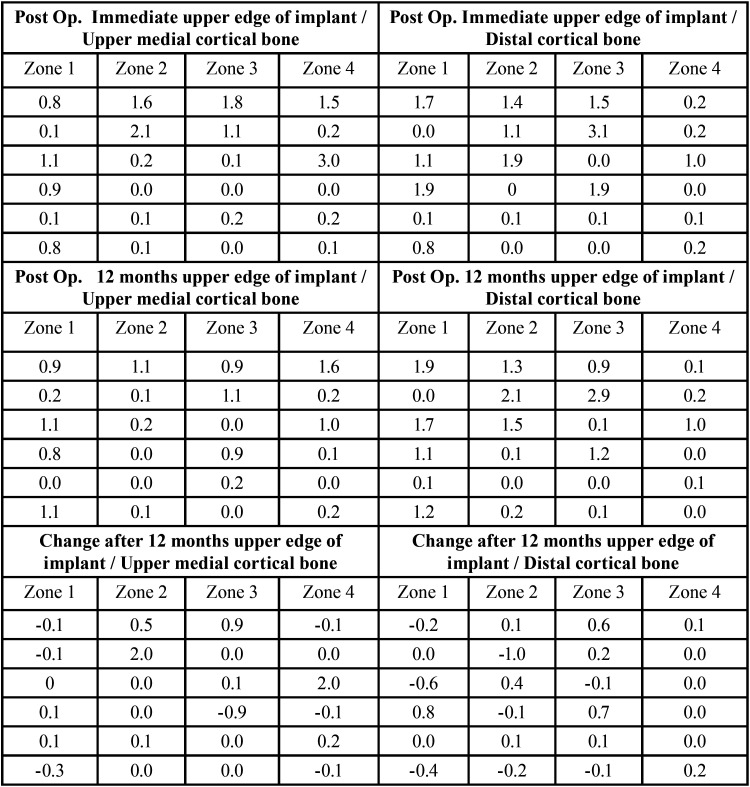
Results after 12-month follow-up in operated zones.

Concerning the mean distances between implants and the anatomical structures in the mandibular zone we can provide the following data: distance between anterior and posterior implants, on each side, was 42.26 mm ± 8.24 and between the anterior ones was 10.81 mm ± 3.00 (Table 1); the distance from the mental nerve to the posterior implants was 24.6 mm ± 6.04 and from the mental nerve to the anterior implants was 17.66 mm ± 5.78; the inter-mental distance was 46.15 mm ± 10.12; the distance to the lower dental nerve, from the upper cortex, in zones 1 and 4 was 5 mm ± 5.28; the distance between the upper and lower cortex in zones 2 and 3 (anterior) was 18.61 mm ± 8.80, whilst in zones 3 and 4 it was 15.03 mm ± 6.07.

## Discussion

In our study we observed that we could obtain predicTable results by placing the implants in anterior sectors and in an internal oblique line. Currently, dental implant placement in anatomical flying buttresses are valid techniques and with a high predictability rate for rehabilitation of patients with large maxillary resorption, due to corticalisation in these zones. The internal oblique line is an anatomical zone which is usually maintained despite large mandibular resorption, and for this reason it is a surgical procedure to be considered for rehabilitation, with implants, for mandibles with severe atrophies.

In 2011, Chang *et al.* (28), described the short implant placement technique in atrophic maxillae, correlating it with the atrophic mandible, and they determined the importance of a prior good imaging study using both conventional panoramic radiographs and computerised tomography studies, including volume tomography (Cone-Beam), as was undertaken with our patients, thus ensuring proper surgical planning.

In 2010, Oh *et al.* (29) published an article stating that wide-diameter dental implant placement together with bicortical fixation can minimise the risk of mandibular fractures, corroborating the results of our study.

Almasri and El-Hakim (30) in a 2012 study, commented that special care must be taken with implants placed in the anterior zone in atrophic mandibles since they can present complications such as mandibular fractures. Likewise, Woltmann *et al.* (31), in their 2011 study, propose placing a rigid internal fixation with locking plates to minimise the risk of fractures in said zone in patients with large resorption. One of our patients presented a mandibular fracture prior to the placement of implants which was properly reduced and then the implants were placed.

In 2015, Boven *et al.* published a systematic review with reference to patient satisfaction with the use of overdentures over implants, obtaining positive results due to the improvement in mastication and the comfort they offer (32). In 2019, Mishra *et al.* published a systematic review with reference to oral health and quality of life in patients with overdentures, resulting in greater retention, stability, comfort, diction and consequently better quality of life for treated patients, and both studies coincide with the type of prosthesis that has been described in this article (33).

All the surgical techniques for bone regeneration in maxillo-mandibular major defects described in this article, have high morbidity, cost, long healing time, complications and discomfort for the patient, and for this reason the internal oblique line technique has been described, in which predictability and treatment success can be seen.

The use of the surgical technique for implant placement in internal oblique line in major mandibular atrophies reduces surgery time, hence the surgery involves lower economic costs, resulting in this procedure being more affordable for patients (mainly older adult patients).

## Conclusions

1. Treatment with this technique is possible without the need for subjecting patients to long and complicated surgical procedures, leading to increased acceptance for the treatment, comfort and post-operative improvement for the patient.

2. On performing only one surgical intervention in which the implants are placed on atrophic ridges without the need to resort to more complex procedures such as grafts, distractions or lateralisations of the mandibular nerve, reduced morbidity for the total procedure is achieved, which will directly lead to a higher success rate for the implants and the patient’s speedy return to normal life.
